# Optimisation of Folate-Mediated Liposomal Encapsulated Arsenic Trioxide for Treating HPV-Positive Cervical Cancer Cells In Vitro

**DOI:** 10.3390/ijms20092156

**Published:** 2019-04-30

**Authors:** Anam Akhtar, Lucy Ghali, Scarlet Xiaoyan Wang, Celia Bell, Dong Li, Xuesong Wen

**Affiliations:** Department of Natural Sciences, Middlesex University, The Burroughs, Hendon, London NW4 4BT, UK; a.akhtar@mdx.ac.uk (A.A.); l.ghali@mdx.ac.uk (L.G.); scarlet.wang@kcl.ac.uk (S.X.W.); c.bell@mdx.ac.uk (C.B.); d.li@mdx.ac.uk (D.L.)

**Keywords:** arsenic trioxide (ATO), liposome, targeted drug delivery, cervical cancer, human papilloma virus (HPV), folate conjugation

## Abstract

High-risk human papilloma virus (HPV) infection is directly associated with cervical cancer development. Arsenic trioxide (ATO), despite inducing apoptosis in HPV-infected cervical cancer cells in vitro, has been compromised by toxicity and poor pharmacokinetics in clinical trials. Therefore, to improve ATO’s therapeutic profile for HPV-related cancers, this study aims to explore the effects of length of ligand spacers of folate-targeted liposomes on the efficiency of ATO delivery to HPV-infected cells. Fluorescent ATO encapsulated liposomes with folic acid (FA) conjugated to two different PEG lengths (2000 Da and 5000 Da) were synthesised, and their cellular uptake was examined for HPV-positive HeLa and KB and HPV-negative HT-3 cells using confocal microscopy, flow cytometry, and spectrophotometer readings. Cellular arsenic quantification and anti-tumour efficacy was evaluated through inductively coupled plasma-mass spectrometry (ICP-MS) and cytotoxicity studies, respectively. Results showed that liposomes with a longer folic acid-polyethylene glycol (FA-PEG) spacer (5000 Da) displayed a higher efficiency in targeting folate receptor (FR) + HPV-infected cells without increasing any inherent cytotoxicity. Targeted liposomally delivered ATO also displayed superior selectivity and efficiency in inducing higher cell apoptosis in HPV-positive cells per unit of arsenic taken up than free ATO, in contrast to HT-3. These findings may hold promise in improving the management of HPV-associated cancers.

## 1. Introduction

Cervical cancer is the second most common cancer in women worldwide and imposes a disproportionately high burden (>80%) on the developing world [1,2]. Since the 1990s, HPV infection has been established as the causal agent of cervical cancer with undisputed epidemiological studies, augmented by molecular technology [3,4]. Further studies have indicated that HPV infection might also be responsible for a subset of anal, vulvar, vaginal, penile, upper respiratory-digestive tract, and head and neck cancers [5,6,7,8]. Therefore, targeting HPV infection has become a priority in managing HPV-associated cancers, and an anti-HPV agent that can specifically be taken up by HPV-infected cells is ideally required to enhance treatment and minimise off-target toxicity to surrounding non-HPV-infected cells and tissues [9].

Our research team, along with others, have shown that ATO, as a therapeutic drug, downregulated HPV oncogene expression and had an inhibitory effect on HPV-infected HeLa cells [10,11]. ATO is a potent clinical agent for the treatment of acute promyelocytic leukaemia and has demonstrated significant clinical success for other haematological malignancies [12,13]. The mechanisms of ATO’s anti-cancer activity has been studied extensively, and various modes of action have been proposed, including the induction of apoptosis [14], mitochondrial toxicity [15,16], and the generation and accumulation of ROS [14,17]. In addition, ATO has been reported to interfere in other cellular events, for instance tubulin polymerisation, DNA repair, and cell cycle progression [16]. 

Clinical response of solid tumours following ATO treatment, however, has been compromised by its severe side effects due to a requirement of much higher dosages for the treatment [18,19]. In vitro studies on HPV-associated cervical cancers have also clearly indicated that ATO is selective in its action only at low concentrations (~2 µM). When the dose was increased to 5 µM, most of the cells were killed from drug-induced toxicity, irrespective of HPV status [11]. 

Therefore, an effective delivery system that can reduce ATO’s off-target toxicity and expand its clinical utility by increasing its accumulation at tumour sites, extending its circulation time in blood and increasing its specificity in targeting HPV-infected cancer cells, is needed. Encapsulating ATO in liposomes is one such strategy that has the potential to improve the drug’s therapeutic index by reducing its side effects and increasing drug concentration in tumours through an enhanced permeability and retention (EPR) effect [20]. In addition, if active targeting strategies are employed, the encapsulated drug can be directed to the target sites with minimal disturbance to the surrounding cells and tissues [21].

Active targeting, also referred to as ligand-mediated targeting, employs the specific affinity of ligands on the surface of the nanoparticles towards specific receptors or surface molecules overexpressed in target cells, tissues, or organs [22,23,24,25,26]. Aided by the EPR effect, which allows the nanoparticle to carry the drug payload to remain in the vicinity of the target cells, active targeting facilitates its cellular internalisation via the specific ligand–receptor interaction [27]. As a result, this approach is increasingly being recognised as an effective strategy to enhance the therapeutic indices of anti-cancer drugs. From our previous findings, FA was selected as the targeting ligand of choice for our target cells, as its corresponding FR was highly expressed on HPV-positive cells while minimally expressed for HPV-negative cell lines. FA conjugation to ATO liposomes thus has the promise to offer the desired selectivity for targeting HPV-infected cells. 

When folate-targeting nanoparticles were evaluated in preclinical settings, they showed promising results as both therapeutic and diagnostic agents [28,29,30,31,32,33]. However, there are quite a few parameters that need careful consideration to design an effective targeting system. Among them, optimisation of ligand density and ligand-conjugating polyethylene glycol (PEG) spacer length is crucial to enhance the uptake and efficiency of a drug-carrying liposomal construct. While a higher ligand density might be desirable to increase the binding avidity between the liposomes and the target antigens, it might also give rise to potential immunogenic concerns, along with the uncertainty of the effect on liposomal stability resulting from a high ligand density [34,35]. 

The importance of PEG spacer length in FR targeting was succinctly established by Lee and Low for KB cells using both targeted and non-targeted liposomes [36]. Liposomes with folate tethered to a long PEG spacer (250 Å) displayed a higher uptake than either non-targeted or targeted liposomes with short spacer length [36]. Additionally, in another study by Gabizon et al. (1999), placing the ligand on a longer PEG spacer length of 3350 Da, as opposed to a 2000 Da PEG layer used for steric stabilisation, increased the uptake of conjugated liposomes substantially [37]. However, it has also been speculated that, if the ligand is conjugated to a longer PEG spacer than the other PEG chains involved in the PEG brush, the extra length of the long spacer might undergo mushroom-like folding, leading to limited exposure of the ligand [35]. Therefore, it might be preferable to have the same length spacer as the surrounding PEG brush [35]. This ambiguity in the literature about the appropriate spacer PEG length requires a thorough investigation for folate conjugation in order to achieve an enhanced drug uptake in a tumour-specific manner.

As a complimentary strategy to EPR, ligand-functionalised nanoparticles offer the potential to further augment the efficacy of nanotherapeutics in treating diseases. Although there have been multiple studies reporting the enhanced uptake by FR-positive cells following folate conjugation to liposomes carrying the therapeutic payload [33,38,39,40], research that focuses on determining the optimal spacer length for FA conjugation on liposomal physicochemical characteristics, inhibitory potency, and in vitro performance on cervical cancer cells with differing HPV status cannot be found. The aim of this study was to assess the effect of ligand spacer length on cellular uptake and inhibitory potency against HPV-positive cervical cancer cells in vitro and to identify the most suitable liposomal formulation for delivering ATO to cervical cancer cells. The uptake and anti-tumour efficacy of the liposomal formulations were examined using two cervical cancer cell lines, HeLa (HPV-positive) and HT-3 (HPV-negative), along with two control cell lines, KB (FR-positive) and A549 (FR-negative).

## 2. Results

### 2.1. Liposome Preparation and Characterisation

**Figure 1:**
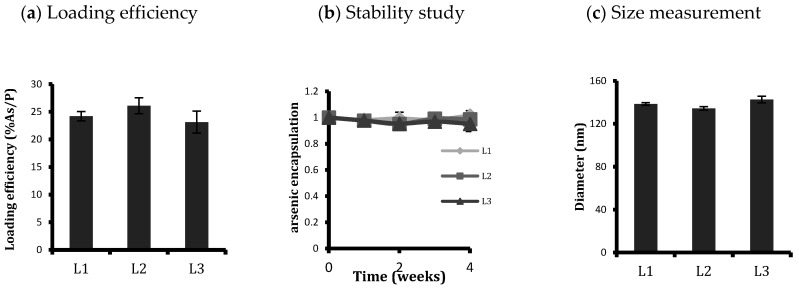
Characterisation of synthesised liposomes: (**a**) loading efficiency (%As/P), (**b**) ATO encapsulated in liposomes over four weeks of storage at 4 °C, and (**c**) diameter of liposomes as measured from Zetasizer with a polydispersity index of around 0.1. Conjugation or length of conjugating PEG spacer does not have any considerable effect on loading efficiency, stability over time in storage, or size of the liposomes. Data are means ± standard deviations of three replicate measurements of at least three independent experiments.

Three labelled unconjugated and conjugated liposomal formulations (L1, unconjugated ATO liposomes; L2, FA-ATO liposomes with 2000 Da PEG spacer; L3, FA-ATO liposomes with 5000 Da PEG spacer) were synthesised, and the concentration of phospholipids, encapsulated arsenic, and nickel were determined by ICP-OES. All of the formulations were found to be efficiently loaded with arsenic, which was co-encapsulated with the transition metal ion Ni; loading efficiency (As/P) is depicted in Figure 1a. The loading efficiency was similar for all the liposomes, irrespective of the presence or absence of FA conjugation. The stability of the liposome suspension was analysed by storing it at 4 °C for a period of 4 weeks and calculating its loading efficiency every week (Figure 1b. Less than 5% arsenic leaked from the liposomes in this time period. All of the liposomal formulations displayed similar storage stability. The mean size of control liposomes was determined by dynamic light scattering on a Zetasizer-Nano ZS as represented in Figure 1c. The DLS size measurement of L1, L2, and L3 were 138.5 ± 1.2 nm, 134.4 ± 1.7 nm, and 142.6 ± 3.1 nm respectively. The polydispersity index for the investigated vesicles ranged from 0.1 to 0.15, indicating a homogeneous population.

### 2.2. Analysing Cellular Uptake by Differing Ligand–PEG Spacer Lengths in ATO Liposomes

It has previously been reported in the literature that spacer length of the conjugated ligand impacts the efficacy of the cellular uptake of the nano-vehicle [41]. To assess this aspect, liposomes were conjugated with FA using a PEG spacer with the same length as the surrounding PEG brush (2000 Da) and a different formulation where FA was conjugated to a PEG spacer with a longer length (5000 Da) than the surrounding PEG brush (2000 Da). Previous studies of folate surface receptor expression on the two cervical cancer cell lines investigated found that HeLa (HPV+) cells positively expressed FRs while HT-3 (HPV-) cells only minimally expressed the receptor. A positive control and a negative control cell line for FR expression, KB and A549, respectively, were also included to validate the robustness of the experiments, with their receptor expression reported in the order of KB > HeLa > HT-3 > A549 cells. Subsequently, the cellular uptake of the conjugated and unconjugated liposomes was investigated with techniques including confocal microscopy, flow cytometry, plate reader analysis, and ICP-MS arsenic quantification.

**Figure 2:**
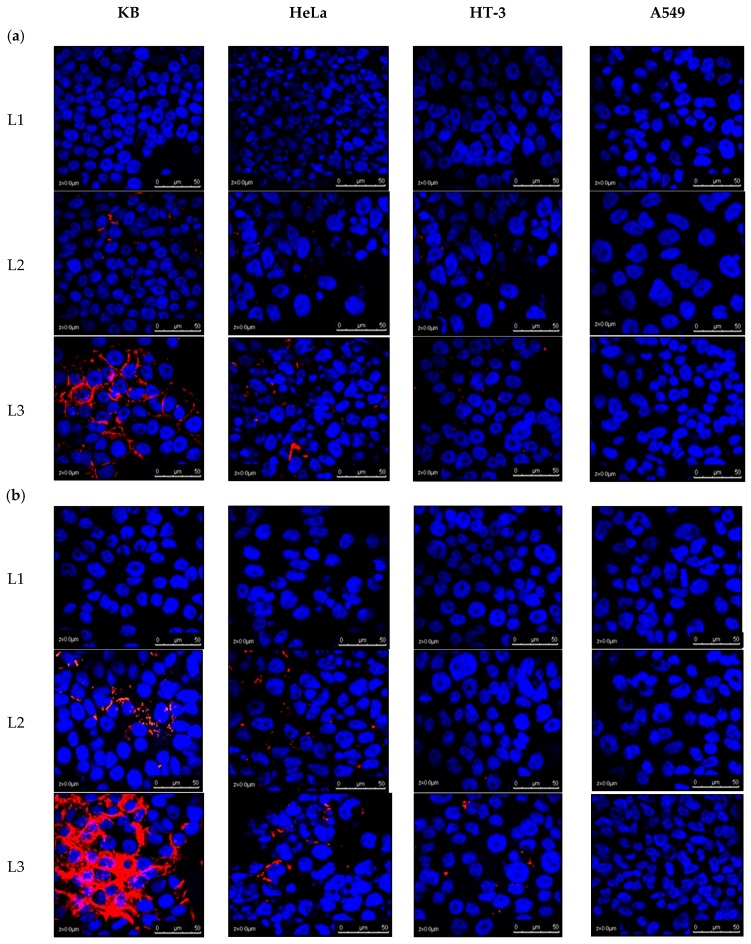

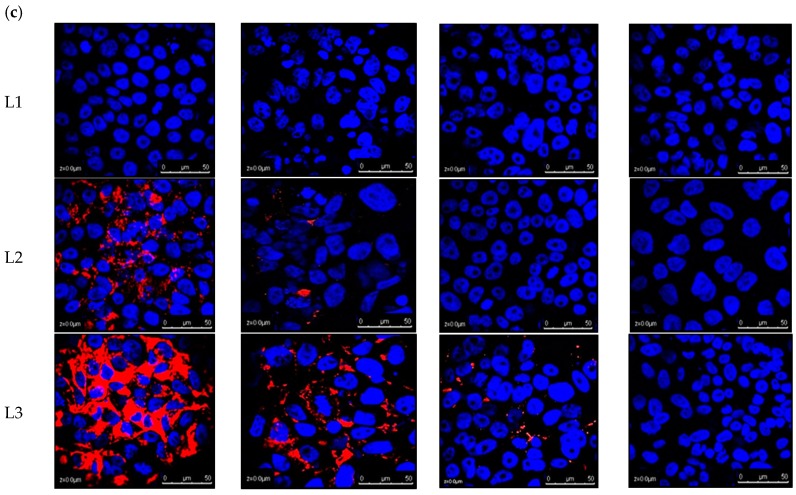
Confocal micrographs showing cellular uptake of DiI-labelled L1, L2, and L3 after treatment of (**a**) 2, (**b**) 6, and (**c**) 24 h. Cells were counterstained with DAPI to reveal the nuclear/DNA location. Among targeted liposomes, L3 were more efficient than L2 in being taken up by cells according to their FR expression, so the uptake in KB was highest followed by HeLa. HT-3 also took up more L3 than L2, while A549 cells had no effect on their uptake from liposomal conjugation. Scale bar: 50 μm.

Confocal laser scanning microscopy (CLSM) allowed for an evaluation of the intracellular fate of liposomes as a function of time for various cell lines. A confocal microscopic visualisation of the cellular association of DiI (1,1′-dioctadecyl-3,3,3′,3′-tetramethylindocarbocyanine-5,5′-disulfonic acid)-labelled liposomes is depicted in Figure 2. The cells were treated for 2, 6, and 24 h, fixed in 4% paraformaldehyde and stained with DAPI (4′,6-diamidino-2-phenylindole) for nuclear imaging. By keeping all the parameters for confocal microscopic visualisation the same (PMT and % laser), it was possible to draw a comparative study of the cellular uptake of liposomes, with or without ligand conjugation.

Liposomes without surface functionalisation showed neither membrane accumulation nor cellular internalisation in any of the cell lines up to 24 h, as evidenced by the lack of liposomal fluorescence (Figure 2). In contrast, ligand conjugation, specifically with a 5000 Da PEG spacer length, mediated an efficient fluorescence uptake in FR-positive cell lines. After 2 h incubation, L3 was found to exhibit high cellular binding, for around 90% of KB cells, 25% of HeLa, and 5% of HT-3, as shown by the red fluorescence around the cell surface. L2 liposomes exhibited minor cellular uptake in KB cells. A549 had no effect of ligand conjugation on its liposomal uptake.

KB cells demonstrated cellular internalisation and accumulation of L3 in their cytosol at a relatively large scale from the time course study, which was shown from the red fluorescence surrounding the DAPI-stained nuclei. L2 was also seen to enter most of the KB cells at 24 h treatment, but to a much lesser extent than L3, which displayed a higher efficiency of L3 for being taken up by the FR-positive cells. A similar trend was repeated for HeLa and HT-3 cells: L3 was more efficient in being taken up by the cells than L2, with L3 entering almost all the HeLa cells by the end of 24 h. The cellular uptake of conjugated liposomes (L2 or L3) in HeLa was always higher than HT-3.

**Figure 3:**
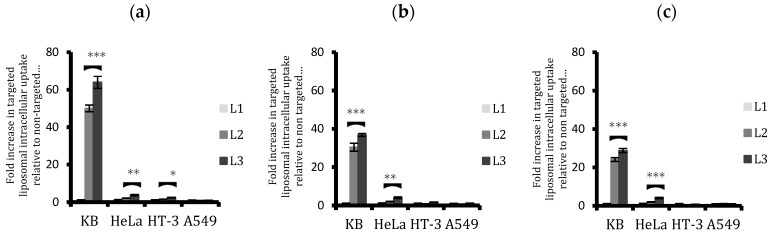
Comparison of the cellular uptake of non-targeted (L1) and targeted liposomes (L2 and L3) by flow cytometry after (**a**) 2, (**b**) 6, and (**c**) 24 h treatment. An unpaired t-test was used to assess any significant difference in the uptakes between the conjugated liposomes, L2 and L3. L3 uptake was significantly higher in both KB and HeLa cells. Data are means ± standard deviations of three replicate measurements of at least three independent experiments. * *p* ≤ 0.05, ** *p* ≤ 0.01, and *** *p* ≤ 0.001.

The results obtained from confocal microscopy analysis were further validated by flow cytometry (Figure 3). The cells from the four cell lines were exposed to fluorescently labelled liposomes for a period of 2, 6, and 24 h. A ratio of cells staining positive for conjugated liposomal (L2 or L3) uptake to non-conjugated (L1) liposomal uptake was calculated for each cell line to determine the efficacy of FA conjugation in enhancing the liposomal cellular internalisation. The results indicated that cellular uptake in FR+ cells increased, even with simple folate conjugation (to a PEG spacer with a length similar to that of the PEG brush)—by about 50 times for unconjugated liposomes in KB cells and to a much lesser extent in HeLa (2.1 times) and HT-3 (1.6 times) cells. When the PEG spacer length was taken into consideration and increased, i.e., from 2000 kD to 5000 kD, this further enhanced the uptake. After 24 h, L3, compared to L2, was taken up 1.2 times more in KB, twice as much in HeLa, and 1.3 times more in HT-3. A549, on the other hand, displayed a similar cellular uptake of liposomes, regardless of ligand conjugation.

**Figure 4:**
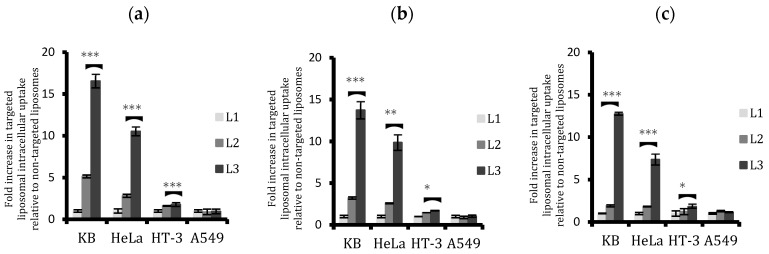
Comparison of cellular uptake of the three liposomal formulations L1, L2, and L3 by the four cell lines with plate reader analysis after (**a**) 2, (**b**) 6, and (**c**) 24 h treatment. L3 uptake was significantly higher than L2 in KB and HeLa. HT-3 cells also displayed a small but significantly enhanced uptake of L3 compared to L2. The difference between targeted and non-targeted liposomes decreased with time. Data are means ± standard deviations of three replicate measurements of at least three independent experiments. * *p* ≤ 0.05, ** *p* ≤ 0.01, and *** *p* ≤ 0.001.

An additional uptake assay was performed in 96 well plates by reading the fluorescence of cells incubated with fluorescent targeted and non-targeted liposomes via a microplate reader. A comparison was drawn of the differential cellular uptake by analysing the ratio of fluorescence of cells incubated with targeted liposomes to non-targeted liposomes followed by blank correction. Results corroborated the findings from confocal and flow cytometry studies as depicted in Figure 4. Conjugated liposomes (both L2 and L3) were taken up in much higher proportions than non-conjugated L1 in KB and HeLa cells, whereas A549 displayed no difference in uptake from ligand conjugation. HT-3 displayed some increase in uptake in the first six hours with L3 treatment after which the difference with L1 tapered off.

L3 liposomal formulation had a significantly higher uptake than L2 for FR-positive cells. They were taken up around 6.7 times more in KB cells and 4 times more in HeLa cells after 24 h. HT-3 also witnessed a 1.5 times higher uptake from L3 than L2, whereas A549 cells remained unaffected in their liposomal uptake from ligand conjugation. In fact, conjugated liposomes were taken up slightly less than the non-conjugated liposomes by a factor of 0.9 in A549 cells. Similar to the flow cytometry results, the difference between the liposomal uptakes with ligand conjugation was reduced when the treatment time was increased to 24 h. This reduction, while being true for all the cell lines investigated, is more evident from 6 to 24 h than from 2 to 6 h. It is also more obvious for KB cells than HeLa cells.

**Figure 5:**
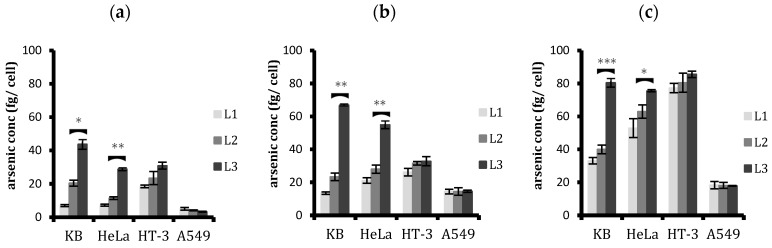
Arsenic concentration per cell as determined by ICP-MS in the four cell lines after (**a**) 6, (**b**) 24, and (**c**) 48 h treatment with the unconjugated (L1) and conjugated (L2 and L3) liposomes. L3 was taken up more than L2 in FR-positive KB and HeLa cells. HT-3 had a higher uptake of liposomes in general, regardless of ligand conjugation. The arsenic concentration increased with time for all cell lines. Data shown is the mean ± SD of three independent experiments. * *p* ≤ 0.05, ** *p* ≤ 0.01, and *** *p* ≤ 0.001.

Cellular liposomal arsenic was quantified with ICP-MS after performing calibrations using arsenic ionic standards and Ga ion as an internal standard. For every experiment performed, we obtained a linear correlation for arsenic with squared correlation coefficients R^2^ >0.997. With this calibration, cellular arsenic was quantified by measuring the total amount of arsenic following digestion of the cells from the four cell lines treated with media only, ATO encapsulating conjugated and unconjugated liposomes for 6, 24, and 48 h. A comparative study of the liposomal treatment was then drawn for cellular arsenic, as depicted in Figure 5.

The results showed that, as the time of incubation increased, the cellular arsenic increased from all treatments for all four cell lines investigated. Quantifying arsenic clearly shows that increasing the ligand–PEG spacer length increases the liposomal uptake by FR-positive cells (Figure 5). After 6 h, L3 liposomes were taken up 2.1, 2.5, 1.3, and 0.8 times more in KB, HeLa, HT-3, and A549, respectively. It was obvious that A549 cells were not affected in their liposomal uptake by an increase in ligand-conjugating spacer length. The same trend was also observed with 24 and 48 h treatment. HT-3, despite showing a slight increase in L3 uptake compared to L2 uptake during the first six hours, reached a similar level with further prolonged treatments.

Corroborating the previous findings, the cellular uptake studies by ICP-MS are consistent with the flow cytometry studies and plate reader analysis in that the difference between the uptakes of targeted and non-targeted liposomes decreases with time in FR+ cells. As KB cells had the highest folate expression on their cell surface, this decline was more pronounced than it was in HeLa cells. On the other hand, HT-3, with minimal expression of cell surface FRs, displayed a slight difference in the first few hours; however, with an increase in time, this difference was nullified, and targeted and non-targeted liposomes were taken up by the cells equally. As for A549 cells, being FR-negative, displayed no difference in the cellular uptakes from ligand conjugation as expected. Regarding the total arsenic content in the cells from the treatment, ICP-MS studies showed that the HT-3 cell line took up more liposomal arsenic in general than all four cell lines regardless of the ligand conjugation, while A549 took up the least.

### 2.3. Analysing Cytotoxicity of Control Empty Liposomes with Differing Spacer Lengths of Conjugated Ligand

**Figure 6:**
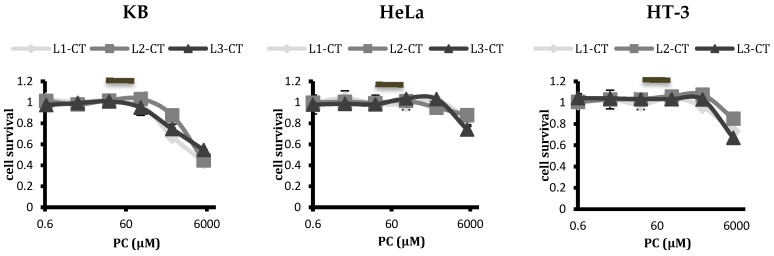
Toxicity studies by MTT assay of the control empty liposomes (L1, L2, and L3) on KB, HeLa, and HT-3 cell lines after 48 h treatment. Increasing the spacer length for ligand conjugation did not result in increased cytotoxicity at the phospholipid concentrations at dilutions used for the treatment. L3 liposomes were non-toxic to all cell lines tested at 48 h, similar to L1 and L2, demonstrating the suitability of L3 as a drug carrier. Points refer to the means, and bars refer to ±SD, where *n* = 3. 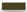 represents the concentrations of phospholipid at dilutions used for treatment of liposomal ATO.

The results above clearly indicated the advantages of using a long PEG spacer to tether FA to the liposomes for enhancing their uptake in FR-positive cells. However, in order to employ them successfully as arsenic nanocarriers to manage cervical cancer, it becomes imperative to investigate any inherent cytotoxicity that they might possess due to the presence of longer PEG spacers. Since our focus is on cervical cancers and the presence or absence of HPV gene sequences, further experiments were carried out on HeLa, HT-3, and KB cells only. The KB cell line was included, since, despite being derived from epidermal mouth carcinoma, it had been contaminated with HeLa cells at some point. Therefore, it was positive for HPV-18 DNA sequences. Empty, unloaded liposomes of various formulations were synthesised and their cytotoxicity analysed on these cell lines via MTT assay at 48 h, as depicted in Figure 6. The dilutions employed for the empty liposomal treatment had their phospholipid concentrations corresponding to ATO liposomes with 5 µM encapsulated ATO concentration. At the dilutions used for treatment and the time period involved, none of the liposomes were toxic for any tested cell lines. With these results in consideration, L3 liposomes, out of the three, prove to be the best liposomal formulation to achieve targeting in FR-positive cell lines.

### 2.4. Quantitative Analysis of Cellular Uptake of Arsenic with Free and Liposomal Arsenic

**Figure 7:**
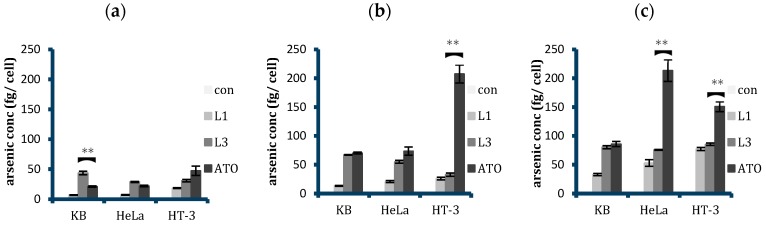
Arsenic concentrations per cell in KB, HeLa, and HT-3 as determined by ICP-MS after (**a**) 6, (**b**) 24, and (**c**) 48 h treatment with only media, unconjugated ATO liposomes L1, FA-conjugated ATO liposomes L3, and free ATO with a 5 µM concentration. Data displayed are mean ± SD from five independent experiments. An unpaired t-test was used to calculate any significant difference in the arsenic intake from L3 and ATO treatments; ** *p* ≤ 0.01. L3 was taken up more than free ATO in KB at 6 h, after which their uptakes reached a similar level when the time of treatment was increased to 24 and 48 h. The cellular arsenic intake from free ATO was higher than L3 in HeLa at 48 h, whereas for HT-3 it became significantly higher after 24 h.

After establishing the superiority of L3 liposomes in the cellular uptake by FR+ cells, they were further investigated in terms of how they compared with ATO when delivered in free form. The cells were treated with L3, L1, free ATO, and media only as control for 6, 24, and 48 h, and their arsenic intake was quantified by ICP-MS (Figure 7).

Results showed that after 6 h of treatment, targeted liposomes L3 were taken up more than free ATO in both KB and HeLa, with cellular arsenic concentration in the order of con < L1 < ATO < L3. Arsenic concentration due to L3 uptake was 2.1 and 1.3 times higher than that from ATO in KB and HeLa cells, respectively. Prolonging the incubation period to 24 h and further to 48 h resulted in reaching a similar level of cellular arsenic concentration from both the L3 and ATO treatments in KB cells. In contrast, with the prolonged incubation time, free ATO was taken up more than L3 in HeLa cells, 1.3 and 2.8 times more at 24 h and 48 h, respectively. In HT-3 cells, free ATO was always taken up more than the liposomal counterparts, both conjugated and non-conjugated, in the time periods tested. The results showed a consistent increase in the cellular arsenic concentration as the incubation time period increased for all the cell lines and the treatments, except the control non-treated sample. However, an unexpected observation was observed with the HT-3 cellular arsenic concentration following free ATO treatment, where instead of increasing from 24 to 48 h, it decreased from 207.4 fg to 150.8 fg per cell.

### 2.5. Selectivity of Targeted Liposomal ATO in Killing HPV-Infected Cervical Cancer Cells

**Figure 8:**
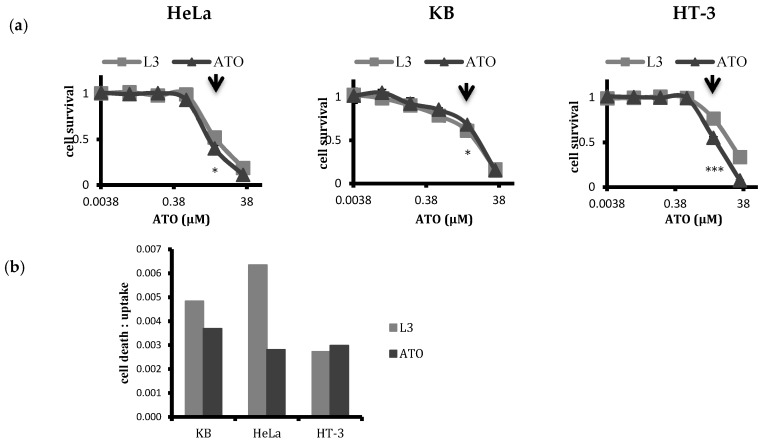
(**a**) MTT test analysis to compare cellular toxicity of targeted liposomal ATO with the free drug on HeLa, KB, and HT-3 cells at 48 h. Points refer to the means and bars refer to ±SD, where *n* = 3. Liposomal encapsulation attenuated drug toxicity to the cells in vitro, except for the KB cell line where L3 liposomes were more toxic than free ATO. (**b**) Ratios of cell death vs. uptake of targeted liposomal and free ATO for KB, HeLa, and HT-3 cells after 48 h treatment. Targeted liposomes induced highest toxicity per unit arsenic uptake in HeLa cells. Targeted liposomes were more effective in inducing cell death when they were corrected for intracellular ATO uptake than free ATO for both FR+ KB and HeLa cells. * *p* ≤ 0.05 and *** *p* ≤ 0.001. 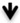 points to 5 µM ATO concentrations that were typically used for treatment.

With differing cellular uptakes of liposomal and free ATO, we further evaluated the cytotoxic response of cervical cancer cell lines of differing HPV statuses (HPV+ HeLa and HPV− HT-3) along with KB (HPV+) to the treatment of ATO delivered either in the free form or encapsulated within the chosen targeted liposomes with 48 h treatment (Figure 8a). The dose–response curve obtained from the MTT assay for the free drug and the liposomal drug revealed that liposomal encapsulation mitigated toxicity, except in the case of KB, where the targeted liposomes L3 induced more cell death than free ATO. At the dilutions used for treatment, i.e., at a 5 µM ATO concentration, the free drug caused more toxicity than L3 in HT-3 and HeLa cells. Cell death–uptake ratios for KB, HeLa, and HT-3 cells (Figure 8b) indicated the level of cell death induced in the cell lines per unit of arsenic taken up by the cells. Targeted liposomal ATO was more effective than the free drug in inducing cell death per unit of arsenic uptake in both folate receptor positive KB and HeLa cell lines, whereas free ATO was more effective in inducing cell death in HT-3 cells. Additionally, between KB and HeLa cells, the liposomal ATO treatment was more effective for HeLa cells than KB cells per unit arsenic.

## 3. Discussion

The efficiency of any actively targeting system is assessed by two important parameters: delivering capacity and targeting specificity of the drug delivery vehicle [20]. The delivering capacity depends on the structure and composition of the nanoparticles [42,43]. In order to optimize an ATO drug nanocarrier for targeting HPV-infected cervical cells, we firstly optimised ATO encapsulating liposomes, with respect to size and charge, for use as delivery vehicles for our target cells [9]. The second parameter, targeting specificity, is mainly determined from the choice of the ligand and how it interacts with the off-target molecules and cells [20]. FA was selected as the targeting agent for folate receptors, which are overexpressed on the surface of targeted HPV cervical cells and minimally expressed on non-HPV cells. In addition, FA has other advantages as an ideal targeting agent, such as being inexpensive and stable with a low molecular weight as well as having simple conjugation chemistry and relative low immunogenicity [27,44,45]. FA has a very high affinity for the FR presented on tumour’s cell surface and is rapidly endocytosed [44]. Since FA is a vitamin required by the eukaryotic cells for the biosynthesis of nucleotide bases, any cargo attached to it remains within endosomes and is then released into cytoplasm rather than shuttled to the lysosome for destruction [27,44]. The folate-targeting of liposomal drug carriers has been reported to increase the therapeutic efficacy in a number of cases, but it requires careful optimisation for effective therapeutic applications for selected tumours [46,47].

The foremost necessity of synthesising an actively targeted nanoparticle is to extend its circulation times, as an increased nanoparticles’ affinity for the target antigens could not compensate for the natural clearance processes in most circumstances [20]. Actively targeted nanoparticles without sufficient steric stabilisation from a PEG layer tend to lose their receptor-binding ability due to non-specific interactions with colloidal proteins [48]. Additionally, since the physical stability of the targeted liposomes without the PEG layer was also not very well explored, in the initial experiments, only the ligand-conjugating PEG spacer was included in the liposomal mix to prepare a targeted ATO delivery system, without the addition of the brush-forming PEG layer. However, this resulted in unstable liposomal formulations, which caused aggregation during the drug loading itself, regardless of the length of the ligand PEG spacer. Adding mPEG_2000_-DSPE to the liposome mix stabilised the targeted formulations and prevented them from aggregating. This might be because surface-grafted PEG chains, even at low concentrations, could provide a sufficient steric barrier to prevent the fusion of liposomal membranes [49].

There was another parameter that required careful optimisation: the ligand density on the surface of the nanoparticle. Thermodynamically, the binding of a ligand to a receptor facilitates the binding of its neighbouring ligands; these multiple interactions lead to a clustering of the receptors, a wrapping of the membrane, and the eventual internalisation of the bound nanoparticle [50]. These multivalent interactions lead to an enhanced avidity of the nanoparticle towards the target cell and prevents its detachment from the cell surface. However, in vitro, a higher ligand density does not necessarily translate to an enhanced cellular uptake [51]. In some cases, it was shown that increasing the ligand density above a certain threshold resulted in saturation of the cooperative effects of the ligands, leading to unfavourable effects on cellular binding [52]. Moreover, the presence of a dense ligand covering on the nanoparticles was also known to make them more prone to being recognised by the cells of a mononuclear phagocytic system (MPS), which consequently led to a loss of their “stealth” surface characteristics [23]. Hence, in order to bypass any such negative effects and to ensure high nanoparticle avidity to the surface receptors, it is necessary to optimise ligand density in the nanoparticles for every particular cancer type.

Since this was an in vitro study, our investigation was primarily focused on the physico-chemical effects of ligand density on liposomal stability and subsequent cellular uptake. There have been reports of ligand molar ratio to range from 0.03 to 0.5% in the literature for folate-targeting liposomes [30,37,53,54,55]. In our experiments, it was observed that using 0.3 mol % DSPE-PEG_2000_-FA and 1.7 mol % mPEG_2000_-DSPE in the liposomal mixture formed stable L2 liposomes. However, the same (0.3 mol %) or higher amount of DSPE-PEG_5000_-FA could not be used to prepare stable L3 liposomes, and this invariably led to the aggregation of liposomes as soon as they were synthesised, despite the presence of a stabilising PEG_2000_ brush layer. After adjusting molar ratios of components, employing 0.1 mol % DSPE-PEG_5000_-FA and 1.9 mol % mPEG_2000_-DSPE yielded stable L3 liposomal formulations. In addition, preliminary confocal uptake studies of L2 liposomes, synthesised with 0.3 mol % or 0.1 mol % DSPE-PEG_2000_-FA, did not show any significant difference between cellular uptakes in HeLa cells. Henceforth, the same ratio (0.1 mol %) was used to prepare both L2 and L3 liposomes for comparison purposes. The instability of L3 liposomes with higher ligand density might be explained by studies where high folate density on the surface of nanoparticles were known to produce dimers, trimers, and quartet self-assembled folate structures, causing an aggregation of nanoparticles, with a longer ligand-conjugating PEG spacer expediting the process [56].

At a molar ratio employed for synthesising targeted liposomes, ligand conjugation had no effect on either the size of the nanoparticles, nor the arsenic loading efficiency and stability (Figure 1). All formulations (L1, L2, and L3) had similar sizes, i.e. around 140 nm, and achieved a high loading efficiency, with an arsenic-to-phospholipid molar ratio of 0.25. Our stability tests showed that all the liposomes, irrespective of ligand conjugation, were homogeneous and stable with a loss of less than 10% arsenic over four weeks of storage at 4 °C (see Figure 1).

Owing to a possibility that PEG brush might mask the targeting abilities of the ligand if it is conjugated to a PEG spacer of equal length, we synthesised L2 liposomes with an FA-PEG spacer of 2000 Da and L3 liposomes with a longer PEG-FA of 5000 Da in length with a surrounding 2000 Da PEG brush. As the previous studies employed FA-PEG3350 [57], we chose to use a longer PEG spacer length of 5000 Da for making targeted liposomes and thus to investigate any increase in cellular uptake along with any inherent toxicity that might arise from employing longer PEG spacers for ligand conjugation. We evaluated the differences in cellular uptake for the liposomal formulations using four different techniques: confocal microscopy, flow cytometry, fluorescence reading via a plate reader, and cellular arsenic quantification via ICP-MS.

Keeping all settings and parameters the same for comparative analysis, confocal microscopic images showed the superiority of using a longer PEG spacer for ligand conjugation (Figure 2). After the initial 2 h incubation, L3 liposomes clearly displayed a superior cell surface attachment in around 80% of FR+ KB cells, in contrast to 10% of KB cells with L2 liposomes. Additionally, around 20% of the HeLa cells showed membrane attachment with L3, but none was observed with either L2 or L1. Some attachment (around 5%) was also seen with L3 in HT-3 cells. A549 (an HPV-negative cell line) showed no cellular association with L3, which demonstrates the superiority of the conjugated ligand to specifically target HPV-positive cells. These results are in accordance with the hypothesis that folate-targeted arsenic loaded liposomes would be efficiently and selectively taken up by FR-positive cells and that, since FR expression in cell lines is in the order of KB > HeLa >> HT-3 > A549, the cellular uptake of targeted liposomes would also follow the same pattern. As the incubation period was increased to 4, 6, and eventually 24 h, KB cells saw a widespread internalisation and accumulation of L3 in their cytosol. L3 was also taken up in almost all of the HeLa cells and around 20% of the HT-3 cells. In contrast, L2, albeit more effective than L1, was visibly less effective than L3 in being taken up by FR+ cells. Based on the confocal images of KB and HeLa cells in particular, the cell surface association of targeted liposomes at 2 h and then the subsequent internalisation and accumulation within the cytoplasm at 24 h was significant, indicating endocytosis.

These results are consistent with results from both the flow cytometric analysis of cellular uptake of fluorescent liposomes and the fluorescence reading from the plate reader, which confirmed the superior and selective uptake of L3 liposomes by FR+ cells (Figure 3 and Figure 4). ICP-MS studies further validated the findings and the results showed that the cellular arsenic content from L3 uptake was almost three times as much as the L2 uptake in KB cells and twice that of HeLa cells after 24 h (Figure 5). This enhanced uptake was, however, not significant in the case of HT-3 cells, owing to the weak FR expression by these cells. FR-negative A549 cells displayed a similar uptake for all the liposomes, irrespective of conjugation or non-conjugation.

This enhanced ATO cellular uptake from L3 liposomes could be explained by folate-receptor-mediated endocytosis. In order to confirm this, future work will be carried out to evaluate drug cellular uptake by blocking FRs with free folates prior to FR-Lip-ATO exposure to the cells. [36]. This would provide useful information for us to understand the uptake mechanism of folate-conjugated liposomes by the target cells. Drugs carried by delivery vehicles could be internalised in the cells via different endocytic pathways, which might prevent the drug from reaching the desired targeted cellular organelle and therefore affecting or even diminishing the therapeutic effect [58]. Of the different pathways proposed for nanomedicinal uptake, clathrin-mediated endocytosis via specific receptor–ligand interaction is the best characterised and has been reported to be the preferred pathway for particles up to 200 nm in size [59]. However, recent studies have suggested that folate-conjugated nanoparticles utilise both clathrin- and caveolae-receptor-mediated endocytosis pathways [60], with the preferred route possibly being dependent on the size of the drug carrier [58]. In one study, it was reported that the uptake of 50 nm nanoparticles followed the clathrin-mediated endocytic pathway, whilst the uptake of 250 nm particles was dominated by caveolae-mediated endocytosis [58]. Since the size of our targeted liposomes was around 140 nm in diameter, they might be predominantly employing the clathrin-mediated endocytic pathway. However, further investigations are needed to confirm this hypothesis.

It was evident from the flow cytometric and plate reader analysis that the difference between the cellular uptake of conjugated and non-conjugated liposomes decreased with prolonging the exposure time (Figure 3 and Figure 4), which might be due to two contributing factors: an increase in non-specific uptake of unconjugated liposomes and a subsequent decrease in the uptake of conjugated liposomes due to a possible saturation of the targeted receptors on the cell surface. This effect was most pronounced for KB cells followed by HeLa. This hypothesis was corroborated after quantifying cellular arsenic via ICP-MS. Results showed that after an initial high surge of targeted liposomal arsenic uptake by FR+ cells, the rate at which they were taken up almost invariably slowed down in comparison with non-targeted liposomes. This led to a bridging of the gap between their uptakes in cells.

Results clearly demonstrated that FA conjugation to liposomes, even to a PEG spacer of similar length as the surrounding PEG brush, was able to selectively target FR-positive cells. However, their targeting efficiency increased considerably when FA was conjugated to a longer PEG spacer, making L3 the logical choice as a delivery vehicle for arsenic to FR rich HeLa and KB cells with HPV infection. However, for it to function as a successful delivery vehicle, any cytotoxicity generated needs to be evaluated. Since we intended to build on our work investigating the therapeutic potential of arsenic against HPV-infected cervical cancers and the time period chosen for our previous studies had been 48 h, all the liposomal formulations were tested for any intrinsic cytotoxicity by incubating empty liposomes for 48 h with HeLa, HT-3, and KB cells (all positive for HPV infection). The dilutions of liposomes tested for toxicity included ones that corresponded with the liposomal concentration with encapsulated 5 µM ATO and that were used for treatment. All the liposomes, including L3, were found to be non-toxic at the dilutions to be used for treatment (Figure 6). This finding was in agreement with the study by Peng et al. (2013), who observed that the conjugated docetaxel delivery systems targeting prostrate specific membrane antigen with a longer spacer length had no significant toxicity in vitro or in vivo in major organs of treated mice bearing a C4-2 tumour xenograft [41]. Kawano and Maitani [61] also reported a higher cellular association of liposomal doxorubicin with a longer ligand–PEG spacer without a significant increase in cytotoxicity.

Consequently, the inherent non-cytotoxicity of L3 liposomes along with their superior targeting abilities led us to select them as an optimised design for a targeted ATO nano-carrier. Further in vitro analysis was also carried out with L3 as the conjugated liposomes, L1 as their unconjugated counterparts, and free ATO on HPV-positive KB and HeLa cells and HPV-negative HT-3 cells.

Cellular uptake studies showed that, usually, arsenic was transported more readily within the cells when the free form was applied (Figure 7). However, owing to the very high FR expression of KB cells, these cells were shown to be most amenable towards folate targeting and consequent liposomal uptake, with L3 liposomes transporting twice as much arsenic in the first six hours of treatment. This trend was also seen with HeLa cells, albeit to a lesser extent. As treatment time increased, however, we reported an equalising of the cellular arsenic concentration between L3- and free-ATO-treated KB cells, whilst an increasing arsenic concentration with free ATO treatment in HeLa cells was surprisingly observed. In HT-3 cells, however, the free form of the drug was always taken up more so than the liposomally delivered forms, with little difference between L1 and L3 uptake.

The cells were further incubated with free and targeted liposomal ATO with a 5 µM concentration for a period of 48 h, and their cytotoxicity was assessed via MTT assay (Figure 8a). As expected, KB cells displayed maximum vulnerability towards L3 treatment, where L3 liposomes induced more cell death than the free drug. In contrast, free ATO generated a higher toxicity than the liposomal formulation for both HeLa and HT-3 cells. The cell death–uptake ratio indicates the cell death induced per unit of arsenic uptake and is an effective means of comparing the efficacy of a treatment on different cell populations. Our results indicated that, for targeted liposomal treatment, this ratio was higher for HPV-positive HeLa and KB cells than it was for HPV-negative HT-3 cells (Figure 8b). Moreover, targeted liposomes were more efficient in inducing cell death than free ATO in FR-positive cells per unit of arsenic taken up, in contrast to HT-3 cells. Interestingly, targeted liposomal ATO was found to be most effective in inducing its response towards HeLa cells, which indicates a certain susceptibility of the cell line towards liposomal treatment, an aspect that warrants further research.

## 4. Materials and Methods

### 4.1. Materials

Folate free RPMI-1640 media, Gibco McCoy’s 5A (modified) medium, foetal bovine serum (FBS), penicillin–streptomycin (5000U/mL), 1,1′-dioctadecyl-3,3,3′,3′-tetramethylindocarbocyanine-5,5′-disulfonic Acid (DiI), nitric acid, Trypan Blue, isopropanol, NaH_2_PO_4_ (sodium dihydrogen phosphate), Na_2_HPO_4_ (disodium hydrogen phosphate), and Trypsin-EDTA were purchased from Fisher Scientific (Loughborough, UK). Soy phosphatidylcholine (PC), cholesterol (Chol), methoxy(polyethyleneglycol)-2000-distearoyl-phosphatidylethanolamine (mPEG-DSPE), 1,2-distearoyl-sn-glycero-3-phosphoethanolamine-N-folate(polyethylene glycol)-2000 (DSPE-PEG_2000_-FA), and 1,2-distearoyl-sn-glycero-3-phosphoethanolamine-N-folate (polyethylene glycol)-5000 (DSPE-PEG_5000_-FA) were purchased from Avanti Polar Lipids (Alabaster, AL, USA). VECTASHIELD Hard Set Mounting Media with DAPI were obtained from Vector Labs (Peterborough, UK). Phosphate-buffered saline (PBS), ATO, nickel acetate, dimethyl sulfoxide (DMSO), thiazolyl blue tetrazolium bromide powder (MTT), Whatman Anotop 0.1 µm syringe filters, and dialysis tubing were purchased from Sigma (Welwyn Garden City, UK). Ham’s F-10 Nutrient Mix, methanol, and dichloromethane were obtained from Thermofisher (Paisley, UK).

### 4.2. Liposome Preparation and Characterisation

Liposomes prepared for this study were labelled with DiI and had compositions as follows: (a) untargeted liposomes (**L1**), PC/Chol/mPEG-DSPE/DiI = 52.5/45/2/0.5 mol %; (b) folate-targeted liposome with a 2000 MW PEG-FA spacer (**L2**), PC/Chol/mPEG-DSPE/DSPE-PEG_2000_-FA/DiI = 52.5/45/1.9/0.1/0.5 mol %; folate-targeted liposomes with a 5000 MW PG-FA spacer (**L3**), PC/Chol/mPEG-DSPE/DSPE-PEG_5000_-FA/DiI = 52.5/45/1.9/0.1/0.5 mol %. Liposomes were prepared as described elsewhere with slight modifications [62]. Briefly, the lipids were dissolved in 1:2 (v/v) methanol/dichloromethane at room temperature. The lipid mixtures were deposited on the side wall of a rotary glass vial by removing the solvent with nitrogen. The dried lipid films were hydrated in 730 mM nickel acetate (Ni(OAc)_2_) aqueous solutions for 1 h with gentle rotation. This process led to the spontaneous formation of multilamellar PEGylated liposomes. The liposome suspension was subsequently subjected to 10 freeze–thaw cycles (freezing in liquid nitrogen for 3 min and thawing in a 37 °C water bath for 3 min and downsized via 0.1 μm Anotop filters. Extruded liposomes were dialysed overnight against 10 mM sodium phosphate buffer at pH 7 to remove unencapsulated Ni(OAc)_2_. The nickel acetate-encapsulated liposomes were then incubated with a 20 mM ATO solution at room temperature with gentle rolling for 5 h. The unencapsulated ATO was further removed by dialysis overnight. The concentrations of phospholipids (P), encapsulated arsenic (As), and nickel (Ni) within the liposomes were determined by an inductively coupled plasma optical emission spectrometer (ICP-OES; Thermo-Scientific iCap 6500 ICP, Stafford, UK), and loading efficiency (As/P ratio) was calculated. Their mean liposomal size was calculated via dynamic light scattering on a Zetasizer-Nano ZS (Malvern Instruments, Malvern, UK). Liposomal stability over a period of a month was assessed by analysing their loading efficiency every week, while liposomes were stored at 4 °C in buffers of pH 7.4.

### 4.3. Cell Culture

HPV-positive and -negative cervical cancer cell lines, HeLa, and HT-3, along with FR-positive and -negative control cell lines, human nasopharyngeal epidermal carcinoma KB, and lung carcinoma A549 cells, respectively (ATCC, Middlesex, UK), were employed in this study. HeLa and KB cells were grown in folate-free RPMI-1640 media, HT-3 cells in McCoy’s 5A (Modified) media, and A549 cells in Ham’s F-10 nutrient mix, supplemented with 10% FBS and 1% penicillin–streptomycin for a minimum of 2 months before each experiment in 75 cm^2^ flasks. The cells were grown in a humidified incubator containing 5% CO_2_ and 95% air at 37 °C until they reached 90% confluence. The following experiments were set up to investigate cellular uptake following liposomal ATO exposure for different time intervals: confocal microscopic visualisation, flow cytometry, spectrophotometer analysis, and quantification of cellular arsenic uptake by ICP-MS studies. Cellular toxicity was investigated via MTT assay.

### 4.4. Qualitative Cellular Uptake Analysis by Confocal Microscopic Visualisation of Liposomal Arsenic

Cells were plated 24–48 h before each experiment, on sterile 22 mm coverslips inside 6-well plates at a density of 5 × 10^4^/mL. Following attachment, the cells were exposed to DiI-labelled liposomes (L1, L2, and L3) for various time intervals at 37 °C in the incubator. The employed liposomal dilution had an arsenic concentration of 18 µmol/L. After the intended time period, the spent media were removed, and cells were washed three times with PBS and fixed with PBS-buffered 4% paraformaldehyde at 25 °C for 8 min. The coverslips were again washed with PBS and mounted on a slide using DAPI containing anti-fade ProLong Gold reagent. The fluorescence emitted from each slide was observed via a fluorescent confocal microscope at 570 nm and 460 nm for DiI and DAPI, respectively (Leica Microsystems, Wetzlar, Germany).

### 4.5. Flow Cytometric Analysis of Liposomal Arsenic Uptake

Cells were seeded at 5 × 10^5^/mL in six-well culture plates and grown overnight before they were exposed to the DiI-labelled liposomal formulations with an arsenic concentration of 18 µmol/L for various time intervals at 37 °C. After the intended time interval, the cells were washed thrice with PBS, trypsinized, washed twice by PBS, and then collected into FACS tubes with 500 µL of PBS. All samples were analysed using FACSCalibur (BD, Oxford, UK). For DiI, the maximum excitation was obtained with an He–Ne laser at 555 nm, and fluorescence emission intensities were observed at 570 nm using an FL-2 filter. For each sample, a minimum of 10,000 cells were collected. Data were analysed using CellQuest Pro software from BD Biosciences. The ratio of cells staining positive with labelled FA-conjugated liposomes (L2 and L3) to cells staining positive for labelled unconjugated liposomes (L1) was calculated to yield an estimate of the differences in the uptake of liposomes of different formulations. All measurements were performed in duplicates from at least three different experiments.

### 4.6. Plate Reader Analysis of Liposomal Uptake by Cells

Cells were seeded at 1 × 10^5^/mL in 96-well plates and allowed to attach overnight. Cells were further exposed to labelled liposomes at arsenic concentration of 18 µmol/L for 2, 6, and 24 h at 37 °C. Following treatment, the cells were washed twice with PBS and the fluorescence measured via BMG LabTech FLUOstar Omega Plate Reader (Bucks, UK). As with flow cytometric analysis, the ratio of cells staining positive with FA-labelled conjugated liposomes (L2 and L3) to cells staining positive for labelled unconjugated liposomes (L1) was calculated and analysed.

### 4.7. Quantitative Analysis of Liposomal Drug Uptake via ICP-MS

Cellular uptake was analysed for different treatments for 6, 24, and 48 h by ICP-MS. Cells from the four different cell lines were first seeded at a density of 1 × 10^6^ cells/flask into sixteen 75 cm^2^ flasks. After a 24 h cell attachment, they were treated with liposomal formulations as mentioned above and a free drug with an arsenic concentration of 10 µmol/L. Following the incubation interval, the cells were washed with PBS, trypsinised, and counted prior to collection in Falcon tubes for further analysis. The cells were lysed by adding 2 mL of nitric acid while vortexing and heating at a temperature of 60 °C for 5 min and were topped up with 8 mL of deionised water. Arsenic concentration was analysed using ICP-MS (Thermo Fisher XSeries2, Paisley, UK) and corrected to the cell number and total volume accordingly.

### 4.8. In Vitro Cellular Cytotoxicity Assay

Cytotoxicity of various liposomal formulations of ATO was determined by the MTT assay as described previously [28]. Briefly, the experiment was set up in a 96-well plate, where the toxicity of control empty liposomes was investigated by taking an initial starting amount containing 0.5 mM of phospholipid concentration and diluting it in 1:10 ratio to another 6 wells. Simultaneously, the toxicity of ATO encapsulating liposomes was calculated by taking the starting amount of 30 µM of ATO concentration, diluting to a 1:6 ratio at a final working arsenic concentration of 5 µM for treating the cells. The wells were seeded with HeLa, HT-3, KB, and A549 at a density of 0.6 million cells per mL and incubated at 37 °C in the humidified incubator for 48 h. After the intended incubation, the spent media was carefully removed and 50 µL of MTT solution was added to each well. Cells were further incubated for 30 min at 37 °C. The MTT solution was then removed and 100 μL of propanol was added per well for at least 30 min incubation at 37 °C to dissolve crystals. The absorbance of this coloured solution was quantified by measuring at a wavelength of 570 nm from BMG LabTech FLUOstar Omega Plate Reader (Bucks, UK).

### 4.9. Statistical Analysis

Statistical analysis described in experimental sections was done using Minitab17. Statistical significance was determined by a two-sample *t*-test. *p* < 0.05 was considered significant. For flow cytometry, statistical analysis was carried out automatically though BD Calibur software provided. For confocal microscopy results, the average number of positively stained cells in a total of six fields for each sample was calculated, and average percentages were recorded.

## 5. Conclusions

The success of a targeted drug delivery system, tailored to the specific cancer under investigation, depends on the thorough optimisation of the nano-carrier’s various parameters. In this study, we synthesised folate-targeted, ATO-encapsulated liposomes and examined the effect of increasing the ligand spacer length on cellular uptake and inhibitory potency against FR-positive cancer cells in vitro, with particular emphasis on HPV-infected FR-positive cervical cancer cells. Folate targeting, particularly when the ligand was attached to a longer PEG spacer, led to an appreciable enhancement in cellular uptake by FR-positive cancer cells. This formulation, along with being efficient and highly specific, had no inherent toxicity of its own at the dilutions used for the treatment, making it suitable to be used as the delivery vehicle of ATO to HPV-positive cervical cancer cells. Future preclinical studies are warranted to elucidate the role of EPR-mediated passive targeting and folate-active targeting in vivo and to investigate nanoparticle clearance/potential toxicity utilising tumour-bearing mice. Our results suggest that further investigation of the molecular mechanisms behind the ATO action delivered via liposomes for targeted liposomal ATO cancer treatment is warranted.

## Abbreviations

%	percentage
µg	microgram
µl	microliter
µM	micromolar
A549	human lung epithelial cancer cell line
As	arsenic
ATO	arsenic trioxide
C	centigrade
Chol	cholesterol
CLSM	confocal laser scanning microscopy
Da	dalton
DAPI	4′,6-diamidino-2-phenylindole
DiI	1,1′-dioctadecyl-3,3,3′,3′-tetramethylindocarbocyanine-5,5′-disulfonic acid
DLS	dynamic light scattering
DMSO	dimethyl sulfoxide
DNA	deoxyribonucleic acid
DSPE-PEG_2000_	methoxypolyethyleneglycol-distearoyl-phosphatidylethanolamine with mPEG MW 2000 Da
DSPE-PEG_2000_-Folate	1,2-distearoyl-sn-glycero-3-phosphoethanolamine-N-folate(polyethylene glycol)-2000 with mPEG MW2000 Da
DSPE-PEG_5000_-Folate	1,2-distearoyl-sn-glycero-3-phosphoethanolamine-N-folate(polyethylene glycol)-5000 with mPEG MW5000 Da
EPR	enhanced permeability and retention effect
FA	folic acid
FACS	fluorescence activated cell sorting
FBS	fetal bovine serum
FITC	fluorescein isothiocyanate
fg	femtogram
FR	folate receptor
Ga	gallium
h	hour(s)
HeLa	human cervical epithelial cells of adenocarcinoma
HPV	human papilloma virus
HT-3	human cervical cancer cell line (HPV-negative)
ICP-MS	inductively coupled plasma-mass spectrometer
ICP-OES	inductively coupled plasma-optical emission spectrometer
KB	human nasopharyngeal cell line contaminated with HeLa cells
kDa	kilodalton
Lipo	liposome
mAb	monoclonal antibody
min	minute(s)
mM	millimolar
mol	molarity
MPS	mononuclear phagocytic system
MTT	3-(4,5-dimethylthiazolyl-2)-2,5-diphenyltetrazolium bromide
NaH_2_PO_4_	sodium dihydrogen phosphate
Na_2_HPO_4_	disodium hydrogen phosphate
Ni	nickle
Ni(OAc)_2_	nickle acetate
nm	nanometre
P	phospholipids
PBS	phosphate buffered saline
PC	phosphatidyl choline
PEG	polyethylene glycol
PMT	photomultiplier tube
SD	standard deviation
UK	United Kingdom
USA	United States of America
v/v	volume by volume

## References

[1] Parkin D.M., Bray F. (2006). The burden of HPV-related cancers. Vaccine.

[2] Schiffman M., Castle P.E., Jeronimo J., Rodriguez A.C., Wacholder S. (2007). Human papillomavirus and cervical cancer. Lancet.

[3] Bosch F.X., Lorincz A., Munoz N., Meijer C., Shah K.V. (2002). The causal relation between human papillomavirus and cervical cancer. J. Clin. Pathol..

[4] Walboomers J.M.M., Jacobs M.V., Manos M.M., Bosch F.X., Kummer J.A., Shah K.V., Snijders P.J.F., Peto J., Meijer C.J.L.M., Munoz N. (1999). Human papillomavirus is a necessary cause of invasive cervical cancer worldwide. J. Pathol..

[5] Nair S., Pillai M.R. (2005). Human papillomavirus and disease mechanisms: Relevance to oral and cervical cancers. Oral Dis..

[6] NeufCoeur P.E., Arafa M., Delvenne P., Saussez S. (2009). Involvement of human papillomavirus in upper aero-digestive tracts cancers. Bull. Cancer.

[7] Shukla S., Bharti A.C., Mahata S., Hussain S., Kumar R., Hedau S., Das B.C. (2009). Infection of human papillomaviruses in cancers of different human organ sites. Indian J. Med. Res..

[8] Steenbergen R.D., de Wilde J., Wilting S.M., Brink A.A., Snijders P.J., Meijer C.J. (2005). HPV-mediated transformation of the anogenital tract. J. Clin. Virol..

[9] Akhtar A., Wang S.X., Ghali L., Bell C., Wen X. (2018). Effective Delivery of Arsenic Trioxide to HPV-Positive Cervical Cancer Cells Using Optimised Liposomes: A Size and Charge Study. Int. J. Mol. Sci..

[10] Um S.J., Lee S.Y., Kim E.J., Myoung J., Namkoong S.E., Park J.S. (2002). Down-regulation of human papillomavirus E6/E7 oncogene by arsenic trioxide in cervical carcinoma cells. Cancer Lett..

[11] Wen X., Li D., Zhang Y., Liu S., Ghali L., Iles R.K. (2012). Arsenic trioxide induces cervical cancer apoptosis, but specifically targets human papillomavirus-infected cell populations. Anticancer Drugs.

[12] Wang Z.Y., Chen Z. (2008). Acute promyelocytic leukemia: From highly fatal to highly curable. Blood.

[13] Zhu J., Chen Z., Lallemand-Breitenbach V., de Thé H. (2002). How acute promyelocytic leukaemia revived arsenic. Nat. Rev. Cancer.

[14] Emadi A., Gore S.D. (2010). Arsenic trioxide—An old drug rediscovered. Blood Rev..

[15] Larochette N., Decaudin D., Jacotot E., Brenner C., Marzo I., Susin S.A., Zamzami N., Xie Z., Reed J., Kroemer G. (1999). Arsenite induces apoptosis via a direct effect on the mitochondrial permeability transition pore. Exp. Cell Res..

[16] Kroemer G., de Thé H. (1999). Arsenic trioxide, a novel mitochondriotoxic anticancer agent?. J. Natl. Cancer Inst..

[17] Platanias L.C. (2009). Biological responses to arsenic compounds. J. Biol. Chem..

[18] Dilda P.J., Hogg P.J. (2007). Arsenical-based cancer drugs. Cancer Treat. Rev..

[19] Liu B., Pan S., Dong X., Qiao H., Jiang H., Krissansen G.W., Sun X. (2006). Opposing effects of arsenic trioxide on hepatocellular carcinomas in mice. Cancer Sci..

[20] Bertrand N., Wu J., Xu X., Kamaly N., Farokhzad O.C. (2014). Cancer nanotechnology: The impact of passive and active targeting in the era of modern cancer biology. Adv. Drug Deliv. Rev..

[21] Allen T.M., Cullis P.R. (2004). Drug delivery systems: Entering the mainstream. Science.

[22] Cheng Z., Al Zaki A., Hui J.Z., Muzykantov V.R., Tsourkas A. (2012). Multifunctional nanoparticles: Cost versus benefit of adding targeting and imaging capabilities. Science.

[23] Kamaly N., Xiao Z., Valencia P.M., Radovic-Moreno A.F., Farokhzad O.C. (2012). Targeted polymeric therapeutic nanoparticles: Design, development and clinical translation. Chem. Soc. Rev..

[24] Koshkaryev A., Sawant R., Deshpande M., Torchilin V. (2013). Immunoconjugates and long circulating systems: Origins, current state of the art and future directions. Adv. Drug Deliv. Rev..

[25] Peer D., Karp J.M., Hong S., Farokhzad O.C., Margalit R., Langer R. (2007). Nanocarriers as an emerging platform for cancer therapy. Nat. Nanotechnol..

[26] Shi J., Xiao Z., Kamaly N., Farokhzad O.C. (2011). Self-assembled targeted nanoparticles: Evolution of technologies and bench to bedside translation. Acc. Chem. Res..

[27] Byrne J.D., Betancourt T., Brannon-Peppas L. (2008). Active targeting schemes for nanoparticle systems in cancer therapeutics. Adv. Drug Deliv. Rev..

[28] Lee R.J., Low P.S. (1995). Folate-mediated tumor cell targeting of liposome-entrapped doxorubicin in vitro. Biochimica et Biophysica Acta (BBA) Biomembranes.

[29] Ni S., Stephenson S.M., Lee R.J. (2002). Folate receptor targeted delivery of liposomal daunorubicin into tumor cells. Anticancer Res..

[30] Pan X.Q., Wang H., Shukla S., Sekido M., Adams D.M., Tjarks W., Barth R.F., Lee R.J. (2002). Boron-containing folate receptor-targeted liposomes as potential delivery agents for neutron capture therapy. Bioconju. Chem..

[31] Sudimack J., Lee R.J. (2000). Targeted drug delivery via the folate receptor. Adv. Drug Deliv. Rev..

[32] Werner M.E., Karve S., Sukumar R., Cummings N.D., Copp J.A., Chen R.C., Zhang T., Wang A.Z. (2011). Folate-targeted nanoparticle delivery of chemo-and radiotherapeutics for the treatment of ovarian cancer peritoneal metastasis. Biomaterials.

[33] Zhao X.B., Lee R.J. (2004). Tumor-selective targeted delivery of genes and antisense oligodeoxyribonucleotides via the folate receptor. Adv. Drug Deliv. Rev..

[34] Sapra P., Allen T.M. (2003). Ligand-targeted liposomal anticancer drugs. Prog. Lipid Res..

[35] Wang M., Thanou M. (2010). Targeting nanoparticles to cancer. Pharmacol. Res..

[36] Lee R.J., Low P.S. (1994). Delivery of liposomes into cultured KB cells via folate receptor-mediated endocytosis. J. Biol. Chem..

[37] Gabizon A., Horowitz A.T., Goren D., Tzemach D., Mandelbaum-Shavit F., Qazen M.M., Zalipsky S. (1999). Targeting folate receptor with folate linked to extremities of poly (ethylene glycol)-grafted liposomes: In vitro studies. Bioconjug. Chem..

[38] Canal F., Vicent M.J., Pasut G., Schiavon O. (2010). Relevance of folic acid/polymer ratio in targeted PEG-“epirubicin conjugates”. J. Control. Release.

[39] Zhang C., Zhao L., Dong Y., Zhang X., Lin J., Chen Z. (2010). Folate-mediated poly (3-hydroxybutyrate-co-3-hydroxyoctanoate) nanoparticles for targeting drug delivery. Eur. J. Pharm. Biopharm..

[40] Zhang Z., Jia J., Lai Y., Ma Y., Weng J., Sun L. (2010). Conjugating folic acid to gold nanoparticles through glutathione for targeting and detecting cancer cells. Bioorg. Med. Chem..

[41] Peng Z.H., Sima M., Salama M.E., Kopečková P., Kopeček J. (2013). Spacer length impacts the efficacy of targeted docetaxel conjugates in prostate-specific membrane antigen expressing prostate cancer. J. Drug Target..

[42] Gu F., Zhang L., Teply B.A., Mann N., Wang A., Radovic-Moreno A.F., Langer R., Farokhzad O.C. (2008). Precise engineering of targeted nanoparticles by using self-assembled biointegrated block copolymers. Proc. Natl. Acad. Sci. USA.

[43] Jiang W., Kim B.Y.S., Rutka J.T., Chan W.C.W. (2008). Nanoparticle-mediated cellular response is size-dependent. Nat. Nanotechnol..

[44] Talekar M., Kendall J., Denny W., Garg S. (2011). Targeting of nanoparticles in cancer: Drug delivery and diagnostics. Anticancer Drugs.

[45] Yu B.O., Tai H.C., Xue W., Lee L.J., Lee R.J. (2010). Receptor-targeted nanocarriers for therapeutic delivery to cancer. Mol. Membr. Biol..

[46] Stephenson S.M., Low P.S., Lee R.J. (2004). Folate receptor-mediated targeting of liposomal drugs to cancer cells. Methods Enzymol..

[47] Reddy J.A., Allagadda V.M., Leamon C.P. (2005). Targeting therapeutic and imaging agents to folate receptor positive tumors. Curr. Pharm. Biotechnol..

[48] Salvati A., Pitek A.S., Monopoli M.P., Prapainop K., Bombelli F.B., Hristov D.R., Kelly P.M., Ãberg C., Mahon E., Dawson K.A. (2013). Transferrin-functionalized nanoparticles lose their targeting capabilities when a biomolecule corona adsorbs on the surface. Nat. Nanotechnol..

[49] Holland J.W., Hui C., Cullis P.R., Madden T.D. (1996). Poly(ethylene glycol)−lipid conjugates regulate the calcium-induced fusion of liposomes composed of phosphatidylethanolamine and phosphatidylserine. Biochemistry.

[50] Mammen M., Choi S.K., Whitesides G.M. (1998). Polyvalent interactions in biological systems: Implications for design and use of multivalent ligands and inhibitors. Angew. Chem. Int. Ed..

[51] Weissleder R., Kelly K., Sun E.Y., Shtatland T., Josephson L. (2005). Cell-specific targeting of nanoparticles by multivalent attachment of small molecules. Nat. Biotechnol..

[52] Elias D.R., Poloukhtine A., Popik V., Tsourkas A. (2013). Effect of ligand density, receptor density, and nanoparticle size on cell targeting. Nanomedicine.

[53] Reddy J.A., Abburi C., Hofland H., Howard S.J., Vlahov I., Wils P., Leamon C.P. (2002). Folate-targeted, cationic liposome-mediated gene transfer into disseminated peritoneal tumors. Gene Ther..

[54] Saul J.M., Annapragada A., Natarajan J.V., Bellamkonda R.V. (2003). Controlled targeting of liposomal doxorubicin via the folate receptor in vitro. J. Control. Release.

[55] Pan X.Q., Wang H., Lee R.J. (2002). Boron delivery to a murine lung carcinoma using folate receptor-targeted liposomes. Anticancer Res..

[56] Ohguchi Y., Kawano K., Hattori Y., Maitani Y. (2008). Selective delivery of folate-PEG-linked, nanoemulsion-loaded aclacinomycin A to KB nasopharyngeal cells and xenograft: Effect of chain length and amount of folate-PEG linker. J. Drug Target..

[57] Chen H., Pazicni S., Krett N.L., Ahn R.W., Penner-Hahn J.E., Rosen S.T., O’Halloran T.V. (2009). Coencapsulation of Arsenic-and Platinum-based Drugs for Targeted Cancer Treatment. Angew. Chem. Int. Ed..

[58] Suen W.L.L., Chau Y. (2014). Size-dependent internalisation of folate-decorated nanoparticles via the pathways of clathrin and caveolae-mediated endocytosis in ARPE-19 cells. J. Pharm. Pharmacol..

[59] Rejman J., Oberle V., Zuhorn I.S., Hoekstra D. (2004). Size-dependent internalization of particles via the pathways of clathrin-and caveolae-mediated endocytosis. Biochem. J..

[60] Turek J.J., Leamon C.P., Low P.S. (1993). Endocytosis of folate-protein conjugates: Ultrastructural localization in KB cells. J. Cell Sci..

[61] Kawano K., Maitani Y. (2011). Effects of polyethylene glycol spacer length and ligand density on folate receptor targeting of liposomal Doxorubicin in vitro. J. Drug Deliv..

[62] Chen H., MacDonald R.C., Li S., Krett N.L., Rosen S.T., O’Halloran T.V. (2006). Lipid encapsulation of arsenic trioxide attenuates cytotoxicity and allows for controlled anticancer drug release. J. Am. Chem. Soc..

